# Clinical Diagnosis: The Bacteriological, Chemical, and Microscopical Evidence of Disease

**Published:** 1890-09

**Authors:** 


### Reviews of Books.

*Clinical Diagnosis: the Bacteriological, Chemical, and Microscopical Evidence of Disease.* By Dr. RUDOLF V. JAKSCH. London: CHARLES GRIFFIN & COMPANY. 1890.

THIS is a translation by Dr. James Cagney from the second edition of a book which has obtained a wide reputation in Germany. It supplies a distinct want in English medical literature, and the practical convenience of such a work is so obvious that we can only wonder that we have hitherto been without anything of the same kind. The object of the book is to give in moderate compass an account of the methods to be followed in the clinical investigation of disease, apart from the actual examination of the patient. Its scope is thus limited to a description of the most approved chemical tests, methods of preparation for microscopic examination and for the detection of micro-organisms, of the naked-eye appearances of the blood and excretions, and of the bodies, normal and abnormal, contained in them. The author indicates the diseases in which the particular morbid changes under discussion are generally found, but wisely abstains from taking away from the practical character of the book by any description of clinical symptoms. Thus we have a handbook to those further methods of clinical examination other than are comprehended under the term physical diagnosis.

The examination of the blood is first considered, then in order that of the nasal and buccal secretions, the sputum, gastric juice, fæces, urine, etc.; and under these heads we have collected in one volume a vast body of information only to be obtained previously by a laborious search through many text-books. The book thus forms a useful work of reference, and in it are embodied the results of much original research, as well as a record of the observations of others. There is an excellent section on bacteriology, in which is given an account of the apparatus necessary and the methods to be followed in the staining and cultivation of micro-organisms, with a short scheme of the plan to be adopted for the recognition of micro-organisms in any fluid from the body. The translator has compiled from the references in the text a bibliography extending over 30 pages, referring for the most part to German authors. Professor Stirling provides an appendix containing matter likely to be useful to English readers; and there is, what is absolutely essential in a book of this kind, a good index.

We think that a useful addition to the end of each section would be a table of the order in which the chief tests, chemical and microscopical, should be employed in the routine examination of the excretion treated of in that section. It would have been more convenient also to have placed under the figures the amount of magnification; in the present arrangement, one has constantly to turn back and hunt up the figure in the list of illustrations at the beginning of the book where the numbers of the ocular and objective used are given. The illustrations are numerous and good; we should single out for special praise the collective views of the microscopic objects commonly found in the vomit and fæces, and the pictures of the urinary crystals. Drawings of the heads of the tænias of the natural size should, we think, have been given, and the pictures of the microscope and of Abbe's condenser in the appendix are now hardly necessary.

On matters of detail we notice that the author considers that further evidence is necessary before the bacillus of tetanus can be considered to be the cause of this disease, and that the discovery of the diplococcus pneumoniæ in the sputum is of little value in diagnosis. Preference is given to the Weigert-Ehrlich and Ziehl-Neelsen fluids for staining tubercle-bacilli; Gibbes' stain, given in the appendix, has proved in our hands unreliable, and it quickly spoils. The author selects as the best test for free hydrochloric acid in the gastric juice benzo-purpurin, and next congo-red and aniline-violet: the general opinion is, we believe, that the two latter are uncertain. The section on the fæces is one of the best in the book, and will be found most useful; there is a good description of the micro-organisms found, and a full account of the methods necessary for the complete detection of the cholera-bacillus; the author calls attention to the frequent presence of hæmatoidin crystals in the fæces. Under the head of the Urine, the tests for albumen recommended are: (1) the nitric acid and heat test, (2) acetic acid and ferrocyanide of potassium, (3) the biuret test, (4) nitric acid in the cold; and it is pointed out that, by applying them in order, it is "possible to form some judgment as to the character of the proteids under investigation." The precautions to be taken in order to ensure a fairly accurate result with Esbach's quantitative test are detailed ; two pages are devoted to the description of Brandberg's modification of Roberts' test for approximate estimation of the amount of albumen—we think unnecessarily. For sugar the author prefers the phenyl-hydrazin test (qualitative); the method given for using Fehling's solution in quantitative testing seems to us to be more troublesome than the ordinary one, without corresponding advantages. Hüfner's apparatus for the estimation of urea by the hypobromite process is not so convenient as others now in use; and a table of the daily excretion of urea according to the different body-weights might have been added with advantage. There are excellent sections on the urine in cases of poisoning, on the detection of certain drugs in the urine, and on the presence of ptomaines in it, and a working classification of the latter bodies is given. The description of the naked-eye appearances of the urine in different states we do not consider so satisfactory.

Dr. Cagney has added some new matter to this English edition ; *inter alia*, an account of Haycraft and Williamson's method of determining quantitatively the alkalinity of the blood, of Fenwick's work on the sulphocyanide of the saliva, and a description of Gowers' hæmacytometer. We cannot conclude without a word of praise for the very excellent manner in which the translation has been accomplished. The book is printed in clear type and neatly bound.

